# Stenosis Length and Degree Interact With the Risk of Cerebrovascular Events Related to Internal Carotid Artery Stenosis

**DOI:** 10.3389/fneur.2019.00317

**Published:** 2019-04-09

**Authors:** Ahmed Mohamed Elhfnawy, Peter U. Heuschmann, Mirko Pham, Jens Volkmann, Felix Fluri

**Affiliations:** ^1^Department of Neurology, University Hospital Würzburg, Würzburg, Germany; ^2^Institute of Clinical Epidemiology and Biometry, University of Würzburg, Würzburg, Germany; ^3^Institute of Diagnostic and Interventional Neuroradiology, University Hospital of Würzburg, Würzburg, Germany; ^4^Department of Neurology, Kantonssptial St. Gallen, St. Gallen, Switzerland

**Keywords:** ischemic stroke, carotid stenosis, carotid atherosclerosis, length of stenosis, degree of stenosis, carotid ultrasound, outcome

## Abstract

**Background and Purpose:** Internal carotid artery stenosis (ICAS)≥70% is a leading cause of ischemic cerebrovascular events (ICVEs). However, a considerable percentage of stroke survivors with symptomatic ICAS (sICAS) have <70% stenosis with a vulnerable plaque. Whether the length of ICAS is associated with high risk of ICVEs is poorly investigated. Our main aim was to investigate the relation between the length of ICAS and the development of ICVEs.

**Methods:** In a retrospective cross-sectional study, we identified 95 arteries with sICAS and another 64 with asymptomatic internal carotid artery stenosis (aICAS) among 121 patients with ICVEs. The degree and length of ICAS as well as plaque echolucency were assessed on ultrasound scans.

**Results:** A statistically significant inverse correlation between the ultrasound-measured length and degree of ICAS was detected for sICAS≥70% (Spearman correlation coefficient ρ = –0.57, *p* < 0.001, *n* = 51) but neither for sICAS<70% (ρ = 0.15, *p* = 0.45, *n* = 27) nor for aICAS (ρ = 0.07, *p* = 0.64, *n* = 54). The median (IQR) length for sICAS<70% and ≥70% was 17 (15–20) and 15 (12–19) mm (*p* = 0.06), respectively, while that for sICAS<90% and sICAS 90% was 18 (15–21) and 13 (10–16) mm, respectively (*p* < 0.001). Among patients with ICAS <70%, a cut-off length of ≥16 mm was found for sICAS rather than aICAS with a sensitivity and specificity of 74.1% and 51.1%, respectively. Irrespective of the stenotic degree, plaques of the sICAS compared to aICAS were significantly more often echolucent (43.2 vs. 24.6%, *p* = 0.02).

**Conclusion:** We found a statistically insignificant tendency for the ultrasound-measured length of sICAS<70% to be longer than that of sICAS≥70%. Moreover, the ultrasound-measured length of sICAS<90% was significantly longer than that of sICAS 90%. Among patients with sICAS≥70%, the degree and length of stenosis were inversely correlated. Larger studies are needed before a clinical implication can be drawn from these results.

## Introduction

Internal carotid artery stenosis (ICAS) causes around one-fifth of ischemic cerebrovascular stroke and has the highest risk of early stroke recurrence in comparison to other stroke subtypes such as cardioembolism or small artery occlusion (1–3). There is a large body of literature showing that the risk of ICAS-related stroke recurrence correlates with the degree of stenosis; ICAS≥70% bears a higher risk compared to ICAS <70% (4). However, there is growing evidence that also low-grade ICAS may lead to ischemic cerebrovascular events (ICVEs) (5, 6). This observation raises the hypothesis that atherosclerotic plaques become unstable because of other characteristics (e.g., plaque composition) and may be finally prone to rupture (“vulnerable carotid plaque concept”) (7). This—in turn–may result in microemboli originating from the surface of plaque. Imaging or pathological biomarkers of plaque, such as fibrous caps, lipid-rich core, and intraplaque hemorrhage have been proposed as indicators of high stroke risk (8).

Duplex ultrasonography is a well-established method to assess the degree of ICAS as well as plaque morphology. Especially, echolucent carotid plaques are often associated with an increased risk for transient ischemic attacks (TIA) and ischemic strokes (9, 10). However, whether the length of ICAS contributes also to the occurrence of stroke is poorly investigated.

In the present study, we aimed to investigate whether (i) the length of ICAS is related to the occurrence of stroke or TIA among patients with sICAS; (ii) there is a relationship between length and degree of ICAS, and whether (iii) plaque echolucency of ICAS <70% is related to stroke or TIA occurrence among patients with sICAS.

## Materials and Methods

### Inclusion and Exclusion Criteria

The raw data supporting the conclusions of this manuscript will be made available by the authors, without undue reservation, to any qualified researcher. We conducted a retrospective cohort study at the Department of Neurology, University Hospital of Würzburg. From January 2011 until September 2016, 121 patients with an ischemic stroke or TIA as well as an asymptomatic or symptomatic ICAS were enrolled in this study. The diagnosis of ICAS was based on ultrasound examination. All patients received electrocardiographic monitoring with atrial fibrillation alarm for a minimum of 24 h. Stroke was diagnosed based on the detection of brain infarction either in the computed tomography or magnetic resonance imaging of the brain and/or the presence of focal neurological signs lasting longer than 24 h (11). Transient ischemic attack (TIA) was diagnosed according to the definition of the American Heart Association/American Stroke Association (AHA/ASA): a transient episode of neurological dysfunction caused by focal brain, spinal cord or retinal ischemia, without acute infarction (12). The ICAS was classified as symptomatic, if the ICVE occurred in the territory of the respective internal carotid artery (ICA). Conversely, the ICAS was classified as asymptomatic, if the ICVE affected the territory of the contralateral ICA or the vertebrobasilar territory. The exclusion criteria were: (1) carotid artery occlusion or pseudo-occlusion because in this case, the measurement of the stenotic length is not possible, (2) patients with merely common carotid artery stenosis without affection of the internal carotid artery, (3) ICAS of 10%, measured according to the hemodynamic criteria of the North American Symptomatic Carotid Endarterectomy Trial (NASCET) (13), because many of these cases could have been easily missed from our records, (4) patients admitted after onset of the ICVE, (5) iatrogenic stroke occurring after carotid endarterectomy or after coronary angiography, (6) no available ultrasound scans of the ICAS, and (7) patients in whom the classification of ICAS as symptomatic or asymptomatic was not possible; this included: (a) patients with brain infarctions ipsilateral to ICAS in the presence of another cardioembolic source according to Trial of Org 10172 in Acute Stroke Treatment (TOAST) (14), lacunar infarction or stroke of other etiology and (b) patients with bilateral brain infarctions, with the main infarction bulk located ipsilateral to the ICAS with further small brain infarction(s) located in the contralateral cerebral hemisphere.

### Measurement of the Degree of Stenosis and Assessment of Plaque Morphology

Ultrasound examination was conducted on a Toshiba AplioXG machine (Toshiba Medical Systems Corporation, Tochigi, Japan) using a 7.5 MHz linear transducer. The degree of ICAS was measured according to the hemodynamic criteria of the North American Symptomatic Carotid Endarterectomy Trial (NASCET) (13). ICAS was categorized into ICAS <70% and ICAS≥70% and further into <90% and 90%. Visual analysis of plaque echolucency on B-mode images was performed by a single observer (AME) using the modified classification proposed by Gray-Weale (15): type 1 (predominantly echolucent), type 2 (mixed echolucent/echogenic), and type 3 (predominantly echogenic).

### Measurement of the Length of Stenosis

**Figure 1:**
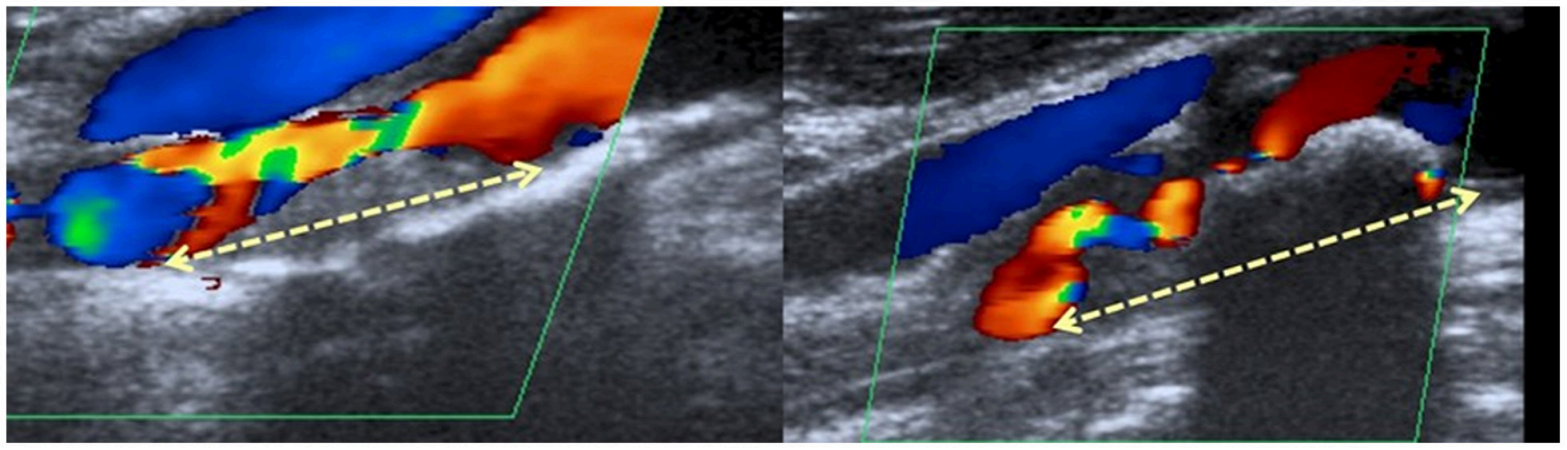
Assessment of the length of internal carotid artery stenosis. A representative example of a duplex scan (B-mode).The length of stenosis was measured from the most proximal to the most distal stenotic area (indicated by a dashed yellow double arrowhead).

The length of ICAS was measured by a single observer (AME) on ultrasound images, which were used as the standard for all statistical analyses. We chose ultrasound as the standard examination because all patients included in this study had available ultrasound images. If the corresponding imaging modalities were available, the length was also measured on magnetic resonance angiography (MRA) and/or digital subtraction angiography (DSA) scans. The two later modalities were used for comparative reasons but not included in the analysis of the statistical relation between the ICVE and the length of ICAS. To measure the length, we chose the projection demonstrating the longest stenotic segment. The length was measured from the most proximal to the most distal part of the stenotic segment as shown in Figure 1. In the ultrasound images, the most proximal and the most distal stenotic segments were defined according to the following criteria: 1. visible narrowing of the vascular lumen, 2. aliasing phenomenon in the proximal end of stenosis. For the distal end, an aliasing phenomenon was accepted, only in the presence of corresponding images showing increased systolic flow velocity denoting the presence of stenosis. In absence of corresponding increased systolic flow velocity, criteria 1 and 3 were used to identify the distal end, because the differentiation between aliasing due to stenosis and post-stenotic flow disturbance was not possible, or 3. the presence of severe calcification, interfering with the visualization of the vascular lumen. In the MRA and DSA scans, criteria one was used to identify the most proximal and most distal stenotic ends.

### Statistical Analyses

Quantitative data were expressed using mean and standard deviation (SD) and/or median and interquartile range, while qualitative data were expressed in absolute and relative frequencies. To check for normality, we used graphical methods (QQ-plot and histogram) and the Shapiro-Wilk test. Univariable statistical tests were conducted using χ^2^ test for categorical data and Mann-Whitney U-test as well as Kruskal-Wallis test for continuous data. Spearman coefficient was used to analyze correlations. For the statistical analyses concerning the length of ICAS, the ultrasound-measured length was used, because all included cases had available ultrasound images. We calculated the sensitivity and specificity of all ultrasound-measured length values using a receiver operating characteristic (ROC)-curve and chose the point with best sensitivity and specificity to represent the cut-off-value differentiating between symptomatic and asymptomatic ICAS. Data were analyzed in SPSS software package version 24 (SPSS, Chicago, IL, USA). *P*-values <0.05 were considered statistically significant.

## Results

**Figure 2:**
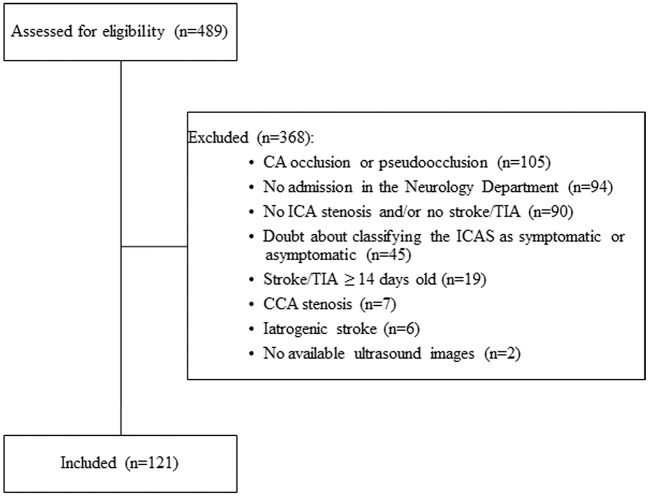
Flow chart showing the included and excluded patients from the current study. CA, carotid artery; CCA, common carotid artery; ICA, internal carotid artery; ICAS, internal carotid artery stenosis; TIA, transient ischemic attack, iatrogenic stroke (5 cases after carotid endarterectomy, 1 case after coronary angiography). Of the 489 patients screened, only 121 patients met our inclusion and exclusion criteria.

Of the 489 screened patients, 121 patients with 159 ICAS met our inclusion criteria. Causes of exclusion are shown in Figure 2 and baseline data in Table 1. The length of stenosis was measured in 134/159 ICAS using ultrasound, in 97/99 ICAS using MRA and in 23/23 ICAS using DSA. The ultrasound scans were used to assess both the degree of stenosis in 157/159 ICAS and plaque echolucency in 149/159 arteries. Ninety-five ICAS (59.7%) were classified as symptomatic and 64 (40.3%) as asymptomatic. The median degree of all measured carotid artery stenoses was 60% (IQR 20%-80%). The median length was 16 (IQR 12–20), 10 (IQR 8–13), and 13 (IQR 10–17) mm in ultrasound, MRA and DSA, respectively. When comparing the imaging modalities with respect to the assessment of the length of stenosis, we found a statistically significant positive correlation between the ultrasound- and MRA-measured length (ρ = 0.35, *p* = 0.002, *n* = 76). A trend for a positive correlation was observed between MRA- and DSA-measured length (ρ = 0.52, *p* = 0.06, *n* = 14). The correlation between the ultrasound- and DSA-measured length was not statistically significant (ρ = 0.46, *p* = 0.1, *n* = 14). Neurosonological examination revealed a significantly higher degree of sICAS with a median of 80 (IQR, 50–90)% compared to 20 (IQR, 20–50)% for aICAS (*p* < 0.001). Overall, the ultrasound-measured length of sICAS and aICAS were similar; median 17 (IQR, 12–20) mm vs. 16 (IQR, 12–19) mm, *p* = 0.66 (Table 2).

**Table 1:** Baseline characteristics of stroke patients with stenosis of internal carotid artery.

**Characteristic**	**Value**
*Age, median (interquartile range), mean (±SD) in years*	74(66–80) 72 (±10)
Male sex, *n (%)*	86 (71.1)
Hypertension, *n (%)*	108 (89.3)
Diabetes mellitus, *n (%)*	42 (34.7)
Smoking status, *n (%)*	
Smoker	28 (23.5)
Ex-smoker	28 (23.5)
Non-smoker	63 (53)
Atrial fibrillation, *n (%)*	8(6,6)^†^
Duration of ECG monitoring in days, *median (IQR)*	5 (3-6)
Type of latest ischemic cerebrovascular event	
Stroke, *n (%)*	88 (72.7)
TIA, *n (%)*	33 (27.3)
History of any previous ischemic cerebrovascular event, either TIA or ischemic stroke, *n (%)*	32 (26)
Previous medications	
Antiplatelets, *n (%)*	49 (56)
Anticoagulants, *n (%)*	18 (10.7)
Statin, *n (%)*	65 (38.7)
NIHSS-score on admission, *median (IQR)*	2 (0-4)
NIHSS on admission, *n (%)*‡	
0	20 (22.7)
1–4	41 (46.6)
5–8	13 (14.8)
9–12	7 (8)
13–17	7 (8)
HbA1c, *median (IQR), mean (±SD) %*	6 (5.6–6.5), 6.3 (±1.2)

*Values are presented as mean median (interquartile range), mean (±standard deviation) or number (%)*.

*ECG, electrocardiography; IQR, interquartile range; TIA, transient ischemic attack*.

† *Atrial fibrillation allowed only for patients with asymptomatic stenosis*.

‡ *TIA patients are not included*.

**Table 2:** Different plaque characteristics among symptomatic and asymptomatic internal carotid stenosis.

	**sICAS (*n =* 95)**	**aICAS (*n =* 64)**	***p*-value**
**PLAQUE ECHOLUCENCY**, ***n (%)***^†^			
Echolucent	38 (43.2 %)	15 (24.6 %)	0.02^‡^
Mixed	14 (15.9 %)	5 (8.2 %)	0.17^‡^
Echogenic	36 (40.9 %)	41 (67.2 %)	0.002^‡^
**SEVERITY OF STENOSIS**^§^			
<70 %, *n (%)*	30 (31.9 %)	55 (87.3 %)	<0.001^||^
≥70 %, *n (%)*	64 (68.1 %)	8 (12.7 %)	
**PLAQUE LENGTH IN DUPLEX**, ***MEDIAN (IQR), mm***			
All degrees	17 (12–20)	16 (12–19)	0.66
ICAS <70 %	17 (15–20)	15 (12 –19)	0.14
ICAS ≥70 %	15 (12–19)	18 (11–20)	0.52
ICAS <90 %	16 (12–19)	18 (15–21)	0.03
ICAS 90 %	13 (10–16)	11, 21^¶^	^¶^

*Values are presented as median (interquartile range) or number (%)*.

*aICAS: asymptomatic internal carotid artery stenosis, IQR, interquartile range, sICAS: symptomatic internal carotid artery stenosis*.

† *In 10 arteries, the available ultrasound images were insufficient to classify plaque echolucency*.

‡ *Statistical analysis for a difference between the mentioned type versus. both other types*.

§ *In two arteries, the available ultrasound images were insufficient to classify the degree of stenosis*.

*^¶^Difference between the distribution of ICAS <70% and ≥70% among sICAS and aICAS*.

*^¶^We had only two available arteries with asymptomatic 90% ICAS, hence statistical analysis was not possible*.

|| *Difference between the distribution of ICAS <70% and ≥70% among sICAS and aICAS*.

### The Relation Between the Length of sICAS and aICAS Among Patients With <70 vs. ≥70% ICAS

**Figure 3:**
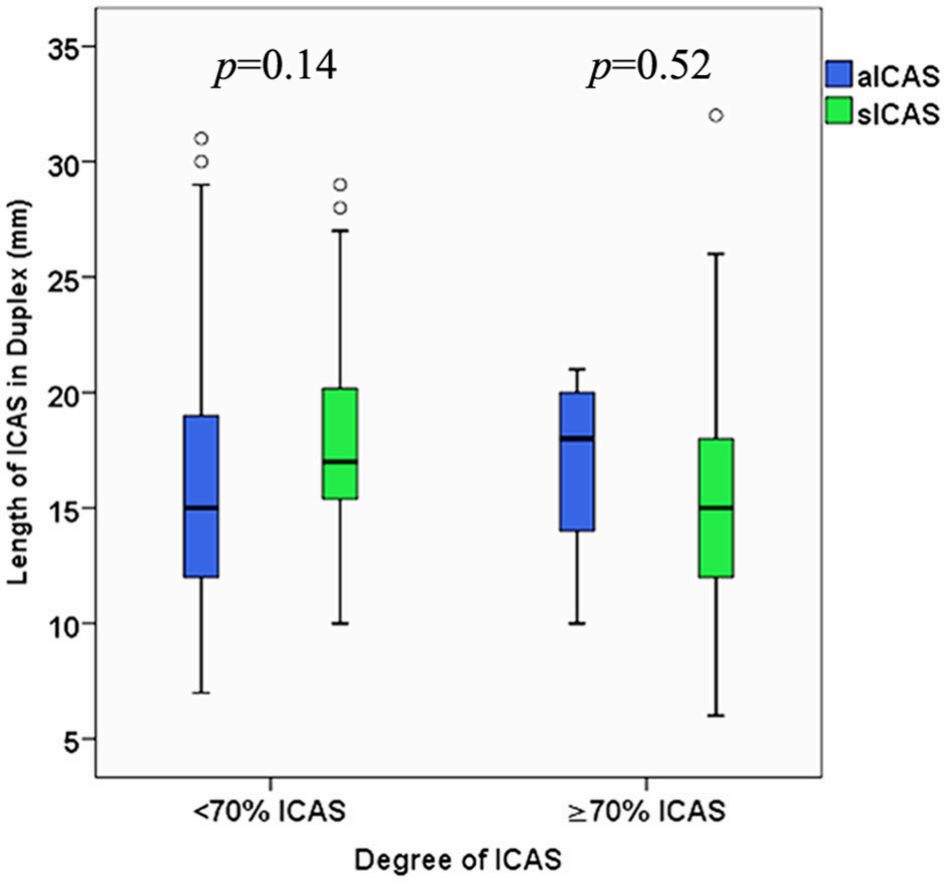
Difference between the ultrasound-measured length of sICAS and aICAS among patients with ICAS <70% vs. ICAS≥70%.

**Figure 4:**
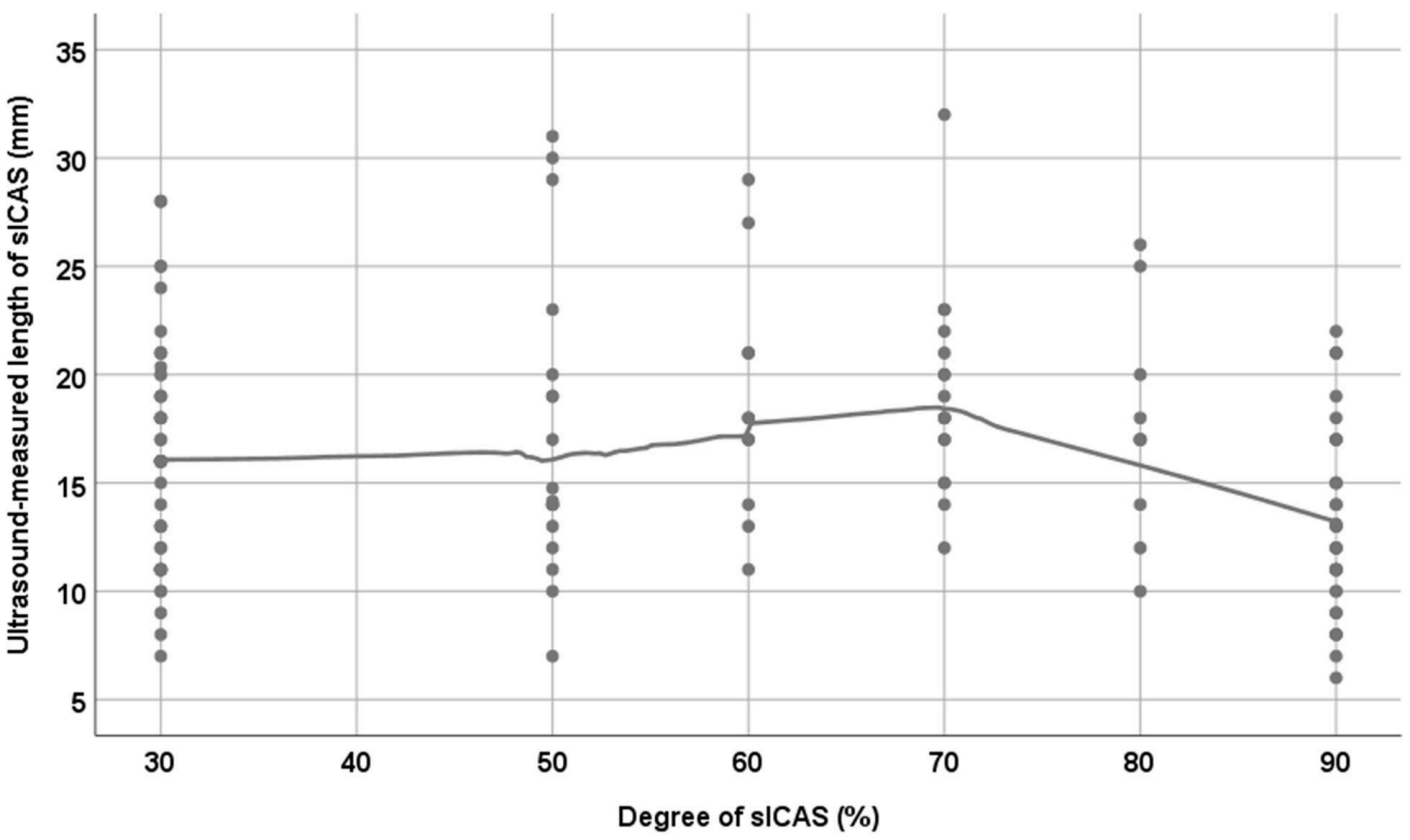
The relationship between the degree and length of sICAS (symptomatic internal carotid artery stenosis). LOESS regression with a smoothing parameter of 0.6 was used to produce the curve. N.b. For technical reasons, 30% was used to represent 20–40% internal carotid stenosis; the NASCET hemodynamic criteria does not differentiate between 20, 30, and 40% internal carotid stenosis.

ICAS was divided according to the degree of stenosis into <70% or ≥70%. The ultrasound-measured length of sICAS<70% was insignificantly longer than that of sICAS≥70%; median 17 (IQR 15–20) mm vs. 15 (IQR 12–19) mm, respectively, *p* = 0.06 (Figure 3). An inverse relationship was observed between the degree measured in duplex and length of sICAS measured by duplex sonography (ρ = –0.39, *p* < 0.001, *n* = 78), MRA (ρ = –0.24, *p* = 0.07, *n* = 60), and DSA (ρ = –0.12, *p* = 0.67, *n* = 15). Further analyses yielded only an inverse correlation among sICAS≥70% (duplex sonography, ρ = –0.57, *p* < 0.001, *n* = 51; MRA, ρ = –0.38, *p* = 0.01, *n* = 44 and DSA, ρ = –0.37, *p* = 0.22, *n* = 13). However, among sICAS <70%, such inverse correlations were no longer observed (duplex sonography, ρ = 0.15, *p* = 0.45, *n* = 27 and MRA, ρ = 0.54, *p* = 0.03, *n* = 16). There were only two available DSA images for sICAS≥70%; therefore, a measurement of the correlation coefficient was not possible. The relation between the ultrasound-measured degree and length of sICAS is shown in Figure 4.

On the asymptomatic side, no statistically significant differences were found regarding the length of stenosis (aICAS <70%, median 15 (IQR 12–19) mm; aICAS≥70%, median 18 (IQR 11–20) mm; *p* = 0.74) (Table 2 and Figure 3). The correlations between the degree, as measured in duplex, and the length of aICAS, as measured in duplex, MRA and DSA were not statistically significant (ρ = 0.07, *p* = 0.64, *n* = 54), (ρ = 0.15, *p* = 0.38, *n* = 36), and (ρ = 0.39, *p* = 0.39, *n* = 7), respectively.

Furthermore, among arteries with ICAS <70%, an ultrasound-measured length of ≥16 mm was observed among sICAS rather than aICAS with a sensitivity and specificity of 74.1 and 51.1%, respectively.

### The Relation Between the Length of sICAS and aICAS Among Patients With <90 vs. 90% ICAS

**Figure 5:**
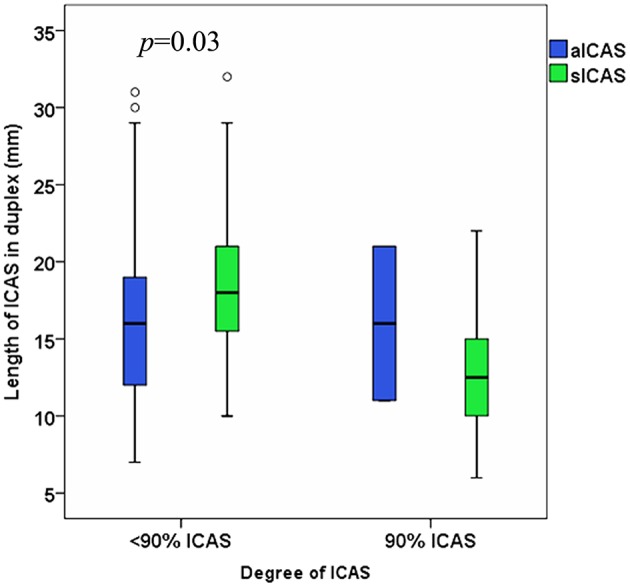
Difference between the ultrasound-measured length of sICAS and aICAS among patients with ICAS <90% vs. ICAS 90%. N.b. We had only two available arteries with aICAS 90%.

Among patients with ICAS <90%, a statistically significant difference between the ultrasound-measured length of sICAS and observed; median (IQR) for sICAS was 16 (12–19) mm vs. 18 (IQR 15–21) mm for aICAS (*p* = 0.03) (Figure 5). Furthermore, a statistically significant difference between the ultrasound-measured length of sICAS<90% and sICAS 90% was observed; median 18 (IQR 15–21) and 13 (IQR 10–16) mm, respectively (*p* < 0.001) (Figure 4).

### Difference of Plaque Echolucency Between sICAS and aICAS

Irrespective of the stenotic degree, sICAS was more often echolucent compared to aICAS (43.2 vs. 24.6%, *p* = 0.02). Echogenic plaques were more associated with aICAS (symptomatic vs. asymptomatic side: 40.9 vs. 67.2%, *p* = 0.002) (Table 2). In patients with ICAS <70%, echolucent plaques were detected in 41.4% of sICAS and in 23.1% of aICAS (*p* = 0.08). In contrast, echogenic plaques were significantly more frequent in aICAS than in sICAS (69.2 vs. 34.5%; *p* = 0.002).

## Discussion

The present study yielded the following main findings: (i) there was a significant inverse correlation between the length and degree of stenosis for sICAS≥70% but neither for sICAS<70% nor for aICAS, (ii) sICAS <70% were insignificantly longer than sICAS>70% (iii) sICAS <90% were significantly longer than sICAS 90%, and (iv) sICAS was more likely associated with echolucent plaques than aICAS.

There is substantial evidence that the degree of ICAS plays a crucial role in the (re)occurrence of ischemic stroke (1). In line with these findings, we also identified a higher degree of ICAS on the symptomatic side.

Furthermore, we found that sICAS was more likely associated with echolucent plaques than aICAS. Similar to our findings, a large systemic review reported that echolucent plaques were more predominant among symptomatic than among asymptomatic carotid stenosis (OR = 3.99, 95% CI = 3.06–5.19) (10). Moreover, echolucent plaques of aICAS were shown to be associated with an increased risk of future ischemic cerebrovascular events (9). Histopathologically, echolucent plaques show increased lipid content, macrophage density, and intraplaque hemorrhage, whereas echogenic plaques have abundant fibrous tissue (16). The risk of ICVE was found to be 6 times higher in relation to the plaques with intraplaque hemorrhage compared to those without hemorrhage, which corresponds to an annual stroke incidence of 17.71% for the former vs. 2.43% for the latter (17). Since echolucent plaques were more often detected on sICAS, this plaque feature might contribute to the previous ICVEs in our patients. This finding remained constant among all stenotic degrees including ICAS <70%.

The inverse relationship between the length and degree of sICAS≥70% might be explained by the early occurrence of ischemic symptoms in patients with high-grade short segment ICAS before the length increases. One might argue that longer plaques result in emboli of atheromatous debris, while more severe stenosis causes more severe flow disturbances, and thus might generate emboli of platelet aggregates. This might also point to different pathogenesis regarding the length and degree of ICAS.

Buon et al. (18) found in young patients with ICAS <50% that plaques of sICAS were significantly longer and more often echolucent compared to aICAS. Interestingly, a plaque length of 12 mm was the cut-off-value in the aforementioned study favoring symptomatic rather than asymptomatic carotid artery with a high sensitivity (86%) and specificity (73%). Of note, the cut-off-value calculated in the present study was higher (i.e., 16 mm). Furthermore, this threshold value yielded a lower sensitivity (74%) and was not specific to discriminate between symptomatic and asymptomatic stenosis (51%). However, Buon and coworkers investigated low-grade stenosis in much younger stroke patients than our cohort and thus a comparison with our data should be considered with caution.

Interestingly, previous authors measured the thickness of carotid plaque for ICAS <50% among patients with embolic stroke of undetermined source (19). The authors observed that thickness of plaque ipsilateral to an ischemic cerebrovascular stroke was more prominent than that on the contralateral side. In the current study, sICAS <70% were insignificantly longer than sICAS≥70% and sICAS <90% were significantly longer than sICAS 90%. It might be postulated that plaque surface area, represented by plaque length in our study and plaque thickness in the aforementioned study, is larger among low-grade sICAS compared to low-grade aICAS. We recommend the inclusion of both parameters, i.e., plaque length and thickness, when investigating the significance of plaques in respect to stroke recurrence in future research.

Carotid revascularization has been shown to be the gold standard treatment for patients with sICAS≥70% in order to decrease the risk of ICVE recurrence (20–22). In patients with moderate sICAS (i.e., 50–69%), carotid revascularization has a marginal beneficial effect and in patients with low-grade carotid stenosis (i.e. 30–49%) it has no effect (21). Future studies addressing the risk of ICVE recurrence related to sICAS <70% should take the length of stenosis in consideration. Moreover, it seems to be reasonable, to measure the length of stenosis, when considering the treatment options for those patients.

Up to now, it remains unclear whether patients with aICAS are more likely to benefit from carotid revascularization or from best medical treatment (23). In the 1990s, CEA has been shown to be superior to Aspirin or deferral of any procedure (24, 25). Later on, it was found that best medical treatment using antiplatelets, statins, smoking cessation, exercise, implementation of a Mediterranean diet and optimal blood pressure management can achieve a significant reduction of cardiovascular events, rate of plaque progression and microembolic signals as shown in the transcranial duplex among patients with aICAS≥60% (26). Moreover, carotid artery stenting was shown to improve the cognition in patients with aICAS (27). In our study, ICAS 90% developed ICVE with yet a short length and ICAS <90% developed their ischemic events after growing in length, hence the length of ICAS represents a new parameter, which should be further examined in large interventional studies to assess whether conservative, CEA or carotid artery stenting is the treatment of choice for these patients.

Several studies have reported that severely stenotic ICAS might remain asymptomatic over years (28). Among patients with aICAS 50–69%, progression of the stenosis was found to be associated with the development of ipsilateral ischemic events (29). Whether the length of stenosis can predict the long-term prognosis or is related to cognitive deficits remains a matter for future research. Furthermore, follow-up of the length of ICAS may be a subject for further studies.

In addition, we found that the length of ICAS was longer in duplex ultrasonography followed by DSA, whereas the length was shortest in MRA. The difference in the length between different examination modalities can be explained by the expected underestimation of the length in MRA and better visualization of the whole length in ultrasound, which can be attributed to the ability to visualize the inner vascular wall demonstrating the whole stenotic plaque in ultrasound examinations. A statistically significant positive correlation was found between the measurement of the length in duplex sonography and MRA and, maybe because of the small number (*n* = 14), only a trend for a positive correlation was found between MRA and DSA. The correlation between the measurement in duplex sonography and DSA (*n* = 14) was not statistically significant. The aforementioned explanation for the difference in the length between different examination modalities, (i.e., measurement of the greatest length of stenosis using duplex sonography, followed by DSA and thereafter MRA), may explain in part the difference in correlations.

### Study Limitations

There are limitations of this study. The non-randomized design of this single-center cross-sectional retrospective study with a convenient sample may have influenced the comparative analysis between aICAS and sICAS, and thus could have resulted in biases. Another limitation of this study is that a single observer (AME) assessed plaque echolucency and length. The reliability and the inter-reader reproducibility of the above-mentioned method, which we used to measure the length of ICAS has to be assessed in further studies. Another disadvantage of our study is that there was a predominance of sICAS in comparison to aICAS, according to our definition, as only patients with stroke or TIA were included.

## Conclusion

Among patients with sICAS≥70%, the degree and length of stenosis were inversely correlated. The ultrasound-measured length of sICAS<70% was insignificantly longer than that of sICAS≥70%, while that of sICAS <90% was significantly longer than sICAS 90%. Echolucent plaque morphology is associated with higher risk for cerebrovascular events. However, these findings have to be confirmed in larger prospective studies.

## Ethics Statement

Data collected within routine clinical care were used. Therefore, no specific approval was needed according to local regulations confirmed by the Ethics Board of the Medical Faculty of the University of Würzburg. Our Ethical Committee was consulted before the conduction of the study and the need for an informed consent was waived because of the retrospective nature of the study.

## Author Contributions

All authors made a substantial contribution to the conception, design, and revision of the draft. AME collected the data, performed the measurements and the statistical analysis and wrote the first draft. PH, MP, JV, and FF supervised the work, provided consultations, and revised the manuscript. All authors were involved in the final approval of the version to be published.

### Conflict of Interest Statement

The authors declare that the research was conducted in the absence of any commercial or financial relationships that could be construed as a potential conflict of interest.
